# Curriculum vitae of CUG binding protein 1 (CELF1) in homeostasis and diseases: a systematic review

**DOI:** 10.1186/s11658-024-00556-y

**Published:** 2024-03-05

**Authors:** Wan‑Jia Qin, Jin-Jin Shi, Ru-Yi Chen, Chang-Yun Li, Yan-Jun Liu, Jian-Fei Lu, Guan-Jun Yang, Jia-Feng Cao, Jiong Chen

**Affiliations:** 1 State Key Laboratory for Managing Biotic and Chemical Threats to the Quality and Safety of Agro-Products, Ningbo University, Ningbo, 315211 Zhejiang China; 2 Laboratory of Biochemistry and Molecular Biology, School of Marine Sciences, Ningbo University, Ningbo, 315211 China

**Keywords:** CELF1, RNA-binding protein, Diseases, Cancer therapy, Inhibitor

## Abstract

RNA-binding proteins (RBPs) are kinds of proteins with either singular or multiple RNA-binding domains (RBDs), and they can assembly into ribonucleic acid–protein complexes, which mediate transportation, editing, splicing, stabilization, translational efficiency, or epigenetic modifications of their binding RNA partners, and thereby modulate various physiological and pathological processes. CUG-BP, Elav-like family 1 (CELF1) is a member of the CELF family of RBPs with high affinity to the GU-rich elements in mRNA, and thus exerting control over critical processes including mRNA splicing, translation, and decay. Mounting studies support that CELF1 is correlated with occurrence, genesis and development and represents a potential therapeutical target for these malignant diseases. Herein, we present the structure and function of CELF1, outline its role and regulatory mechanisms in varieties of homeostasis and diseases, summarize the identified CELF1 regulators and their structure–activity relationships, and prospect the current challenges and their solutions during studies on CELF1 functions and corresponding drug discovery, which will facilitate the establishment of a targeted regulatory network for CELF1 in diseases and advance CELF1 as a potential drug target for disease therapy.

**Graphical Abstract:**

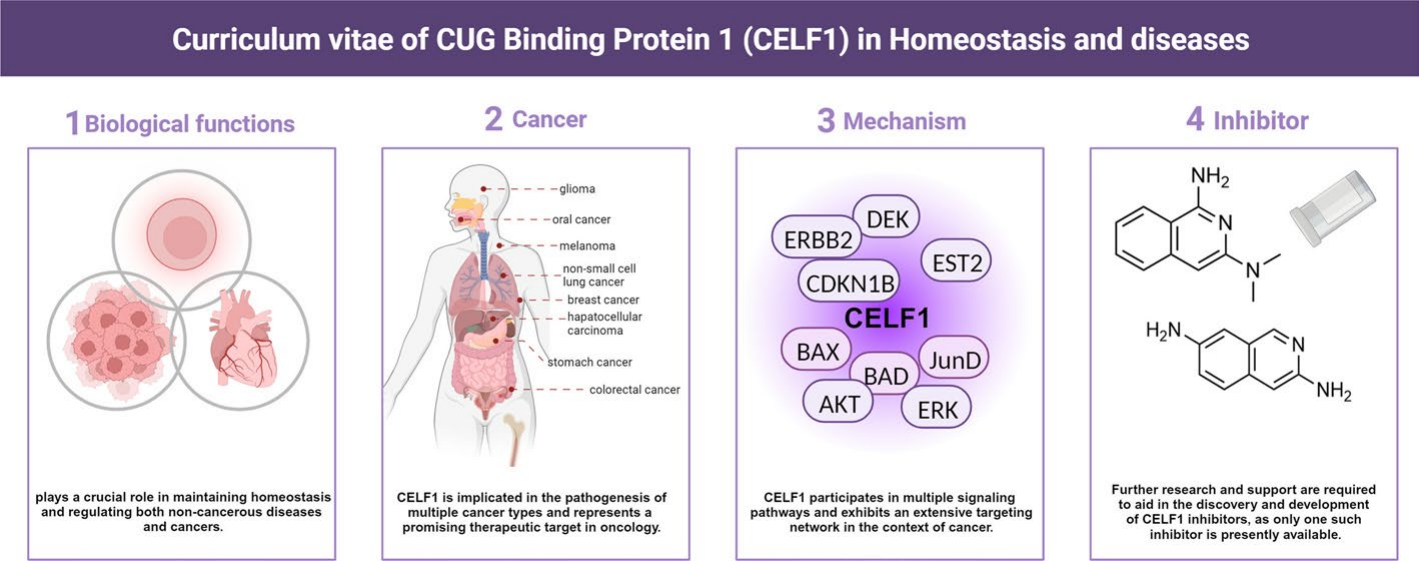

## Introduction

RNA-binding proteins (RBPs) are generally considered as proteins that bind RNAs and alter the fate or function of the binding RNAs through their one or more RNA-binding domains (RBDs) [1]. To date, there are approximate 1542 human RBPs identified based on high-throughput proteomics [2]. RBPs are engaged in interactions with both double-stranded and single-stranded RNA, resulting in the formation of ribonucleic acid–protein complexes, which serve as crucial mediators in the post-transcriptional regulation of gene expression. RBPs possess either singular or multiple RBDs, enabling a single RBP to interact with numerous transcripts, ranging in the hundreds or even thousands. RBPs can be divided into ~ 50 different families based on differences in their RBDs [1]. In addition, RBPs are also classified based on their functional roles in post-transcriptional regulation such as RNA editing, RNA modification, RNA splicing, RNA polyadenylation, RNA translation, nuclear transport, and RNA degradation, etc. [3]. Mounting studies have showed that RBPs mediate maintaining cellular homeostasis and their aberrant expression leads to varieties of diseases via modulating their mRNA targets by specific protein–mRNA complexes [1]. In a word, RBPs assume a pivotal role within the intricate landscape of RBP-RNA regulatory networks implicated in disease progression, notably across diverse cancer types.

The CUG-BP, Elav-like family (CELFs), initially identified as CUG-BP, represent a ubiquitous class of RBPs found in both animal and plant species [4]. The structures, expression profiles, and functions of CELF proteins exhibit a high degree of conservation across different species. Based on the comprehensive analysis of sequence similarity, the CELF family can be classified into two distinct subfamilies. The first subgroup encompasses CELF1 and CELF2 with a protein sequence similarity rate of 76%. The second subgroup comprises CELF3-6, where the protein sequences of these four members exhibit approximately 64% homology among themselves but only 44% homology with either CELF1 or CELF2 [5]. The CELF protein family has been demonstrated to participate in two prominent biological processes localized within the nucleus or cytoplasmic compartments. In the nucleus, CELF proteins have the capability to directly bind to precursor mRNA introns, thereby facilitating appropriate splicing events. Within the cytoplasm, these proteins exert their influence on mRNA deadenylation, stability, and translation by interacting with the 5' and 3' untranslated regions (UTRs) of mature mRNA molecules [6, 7]. CELF1 is an RBP belonging to the CELF family and it fulfills its function in various disease contexts via binding to GU-rich elements within their partner mRNAs, and thereby modulating mRNA splicing, translation, and attenuation, etc. [8, 9]. CELF1 is involved in many body homeostasis such as development of embryonic and heart, differentiation of bone and adipose tissue, as well as formation germ cells [10–12]. The aberrant expression of CELF1 leads to multiple diseases such as myotonic dystrophy type 1 (DM1) [13], myocardial hypertrophy [14]. Extensive genomic and transcriptomic analyses have revealed substantial changes in mutational profiles, copy number variations, and mRNA expression levels of various RNA-binding proteins in numerous tumor types [1]. While significant research has been dedicated to understanding the involvement of CELF1 in oncogenesis, there is a current shortage of studies that provide detailed information regarding the construction of its downstream target network and its functional dynamics. However, recent investigations have shed light on the complex regulatory mechanisms underlying CELF1 in different types of physiological and pathological processes. These findings present promising opportunities for the development of targeted therapeutic interventions within the field.

## Evolutionary conservation of CELF1

### The phylogenetic analysis of CELF1

**Fig. 1:**
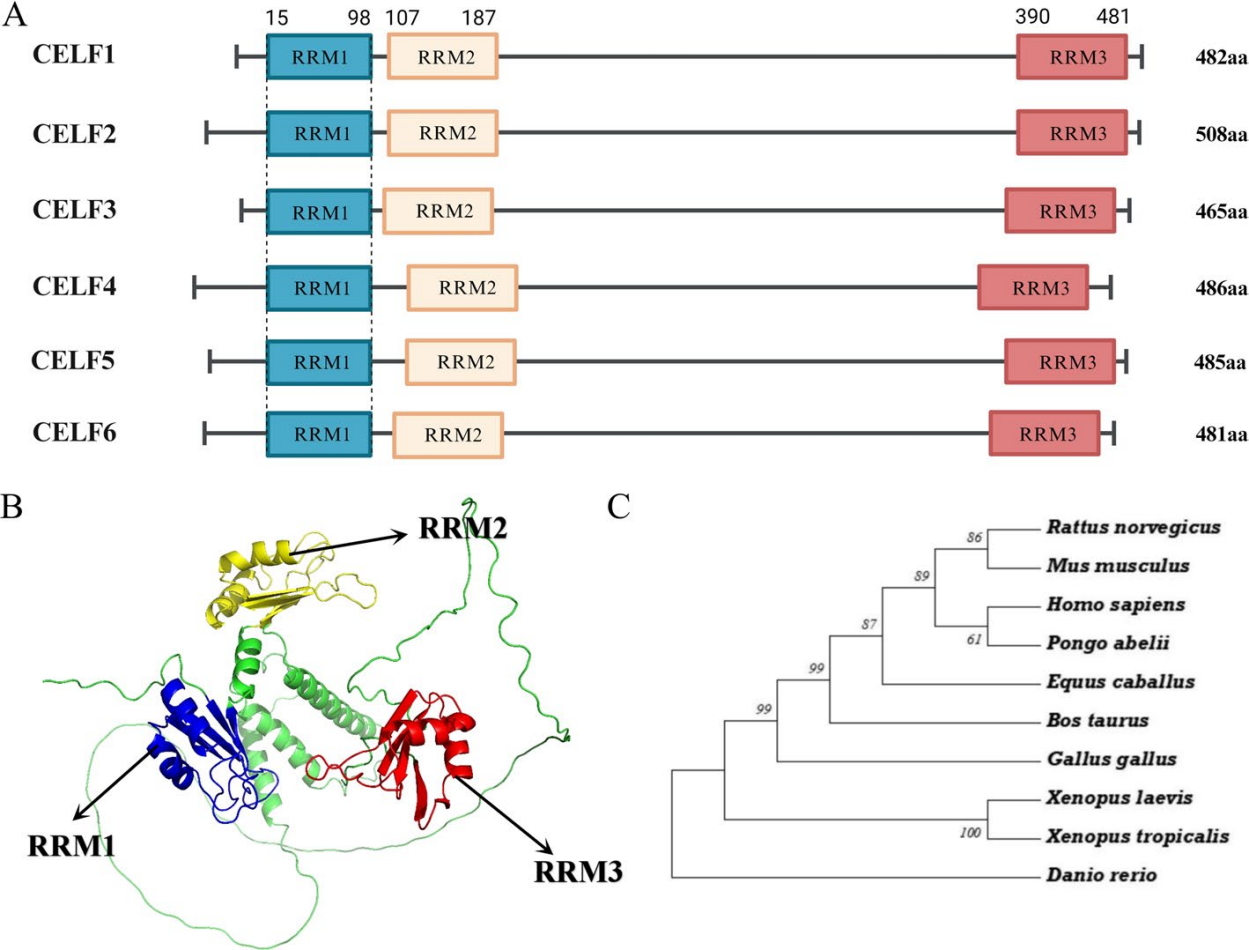
Structural and phylogenetic investigation of CELF1. **A** Comparative analysis of the structural characteristics among six genes within the CELF family. **B** The structural characteristics of CELF1 were determined using AlphaFold, with distinct domains identified and color-coded as follows: the RRM1 domain (amino acid residues 15–98), the RRM2 domain (amino acid residues 107–187), and the RRM3 domain (amino acid residues 390–481). **C** A phylogenetic examination of the amino acid sequence of CELF1 was conducted utilizing MEGA version 7. The value at the node indicates the percentage of trees supporting the specific grouping following bootstrapping

CELF1 shows evolutionary conservation as a transcriptional regulator across multiple species, spanning from *Xenopus laevis* to *Homo sapiens* (Fig. 1C). Phylogenetic analysis has demonstrated that CELF family members share a conserved motif structure across diverse species, revealing CELF1 and its family members play important roles in gene regulation throughout evolution (Fig. 1A). In human genome, the *CELF1* gene is located on chromosome 11 and involved in various biological processes.

### Phylogenetic analysis and chromosome localization of CELF1

The phylogenetic tree analysis revealed the evolutionary conservation of CELF1 across species, spanning from *Xenopus laevis* to *Homo sapiens* (Fig. 1A). The homologous motif analysis using the MEME Suite database (https://meme-suite.org/meme/doc/meme.html) also showed that members of the CELF family exhibit consistent motif structures, indicating shared sequence characteristics amongst these proteins (Fig. 1C). In addition to their potential involvement in tissue-specific developmental processes, CELF1 and CELF2 have also been implicated in various cellular processes, including RNA metabolism, alternative splicing, and translation regulation. The distinct nuclear and cytoplasmic functions mediated by these proteins are likely tailored to the specific requirements of various tissues and cellular contexts [15]. According to the Genecards database (https://www.genecards.org/), the genetic locus of CELF1 in the human genome is situated on chromosome 11. The estimated genome sequence length for this locus is approximately 99,637 nucleotides. This genetic region includes a total of 15 exons, which encode a canonical protein product likely produced by normal splicing and five other isoforms produced by alternative splicing.

## Domain structure of CELF1 protein

The CELF1 protein consists of three conserved RBDs called RNA recognition motifs (RRM). RRM1 and RRM2 are located close to each other at the amino terminal, while RRM3 is positioned near the carboxyl terminal region of the protein. The junction region between RRM2 and RRM3 is situated between these two RNA recognition motifs (Fig. 1B) [16]. The CELF family encompasses all three domains, and certain junctions display specific amino acid sequences that facilitate binding (Fig. 1A). Edwards et al. employed gel blockade, filtration techniques, isothermal titration, and nuclear magnetic resonance experiments to investigate the RNA recognition properties of the first two RRMs of CELF1. Their findings indicate that RRM1 of CELF1 has a broad binding affinity towards UGU and CUG repeats, as reflected in the similar chemical shift perturbations observed for both motifs. Conversely, RRM2 of CELF1 displays a higher specificity towards UGUU motifs compared to CUG motifs [17, 18].

## Biological functions of CELF1

**Fig. 2:**
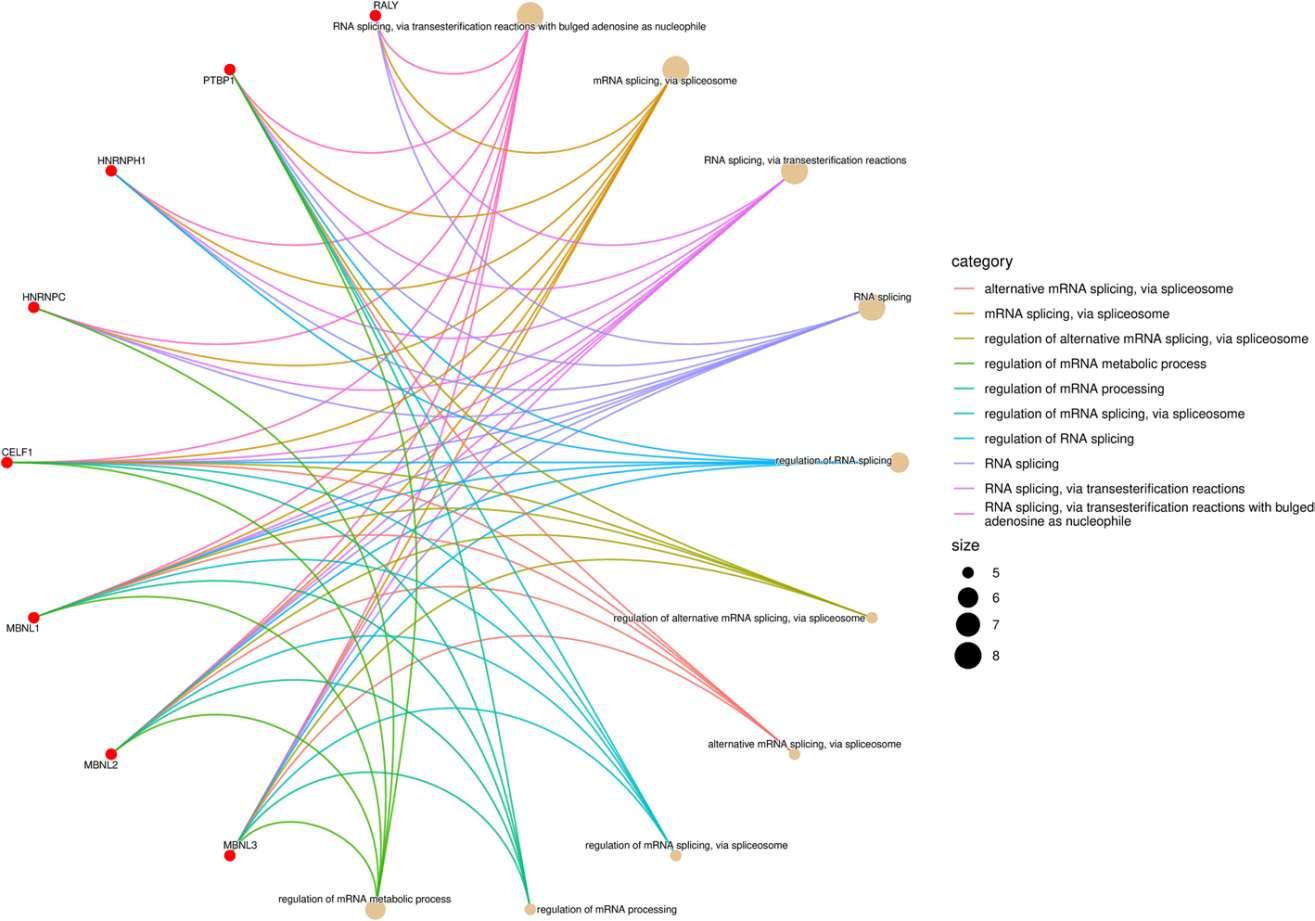
The primary molecular functions, biological processes, cellular components, and KEGG pathways associated with CELF1 interactors were identified. These interactors were sourced from DAVID (https://david.ncifcrf.gov) and STRING database (http://string-db.org). Heatmap was plotted by https://www.bioinformatics.com.cn (last accessed on 10 Nov 2023)

**Fig. 3:**
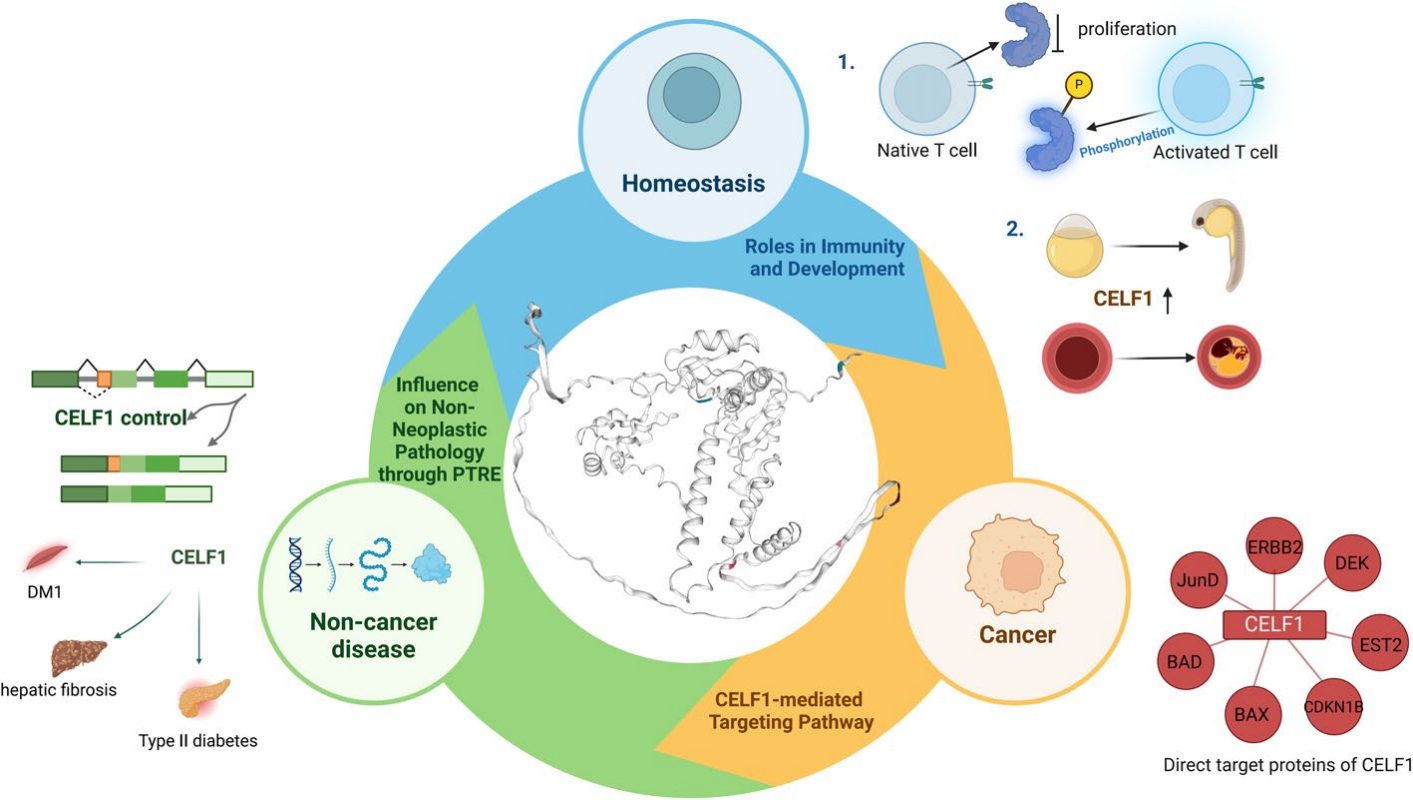
Biological function of CELF1. This diagram illustrates the functional involvement of CELF1 in homeostasis, non-cancerous diseases, and cancer

The functional attributes of RBPs, including CELF proteins, are conserved across diverse species. Prior investigations in model organisms have provided evidence for the involvement of CELF proteins in both gametogenesis and zygotic development. Within the CELF Protein family, the CELF1-2 subfamily is prominent in the regulation of key physiological processes, such as heart functioning, muscles development, and nervous system functioning, whereas the CELF3-6 subfamily appears to have a greater propensity for governing neural activity [19]. Bioinformatics analysis has revealed that CELF1 primarily interacts with proteins involved in splicing processes (Fig. 2). Splicing is an important mechanism by which RNA molecules are processed, allowing for the generation of different protein isoforms from a single gene. By participating in splicing regulation, CELF1 is involved in maintaining homeostasis and exerting its influence on immune responses and developmental processes (Fig. 3).

### CELF1 activates immune cells

The CELF protein plays a crucial role in the gene regulatory network responsible for controlling how immune cells response to external stimuli. It also orchestrates the activation processes of immune cells. In quiescent T cells, CELF1 acts to inhibit the splicing of transcripts related to cellular proliferation. Upon T cell activation, the phosphorylation of CELF1 is occurs, lead to a decrease in its binding affinity of CELF1 towards the target transcript. This phosphorylation-mediated event promotes the stabilization and accumulation of transcripts associated with activation and proliferation, ultimately resulting in the activation of immune cells [20].

### CELF1 contributes to embryonic development

CELF1 has been found to be essential for gametogenesis and embryonic development in various animal models, including mice, Xenopus, and zebrafish. The deletion of *CELF1* in mice leads to deficiencies in gametogenesis [21], while suppression of CELF1 in *Xenopus* in abnormal gametogenesis [19, 22]. In addition, the deletion of CELF1 in zebrafish impairs organogenesis within the endodermal tissue by attenuating the growth and migration of endoderm cells during embryonic gastrula formation [23]. These observations emphasize the critical role of CELF1 in regulating RNA localization and gene expression during early development and provide valuable insights into the molecular mechanisms underlying gametogenesis and embryonic development.

### CELF1 plays a role in myocardial development

The reduced expression of CELF protein in mice has been shown to result in cardiac dysfunction, myocardial hypertrophy, dilated cardiomyopathy, and premature mortality among young individuals. These observations underscore the importance of CELF in regulating cardiac function through alternative splicing dysregulation [24, 25]. Studies have also revealed that CELF1 is involved in normal myofibrillar formation and morphogenesis during embryonic heart development in chickens and Xenopus [26]. Furthermore, aberrant expression of CELF1 has been implicated in the development of cardiac hypertrophy [27].

## CELF1 and related diseases

**Fig. 4:**
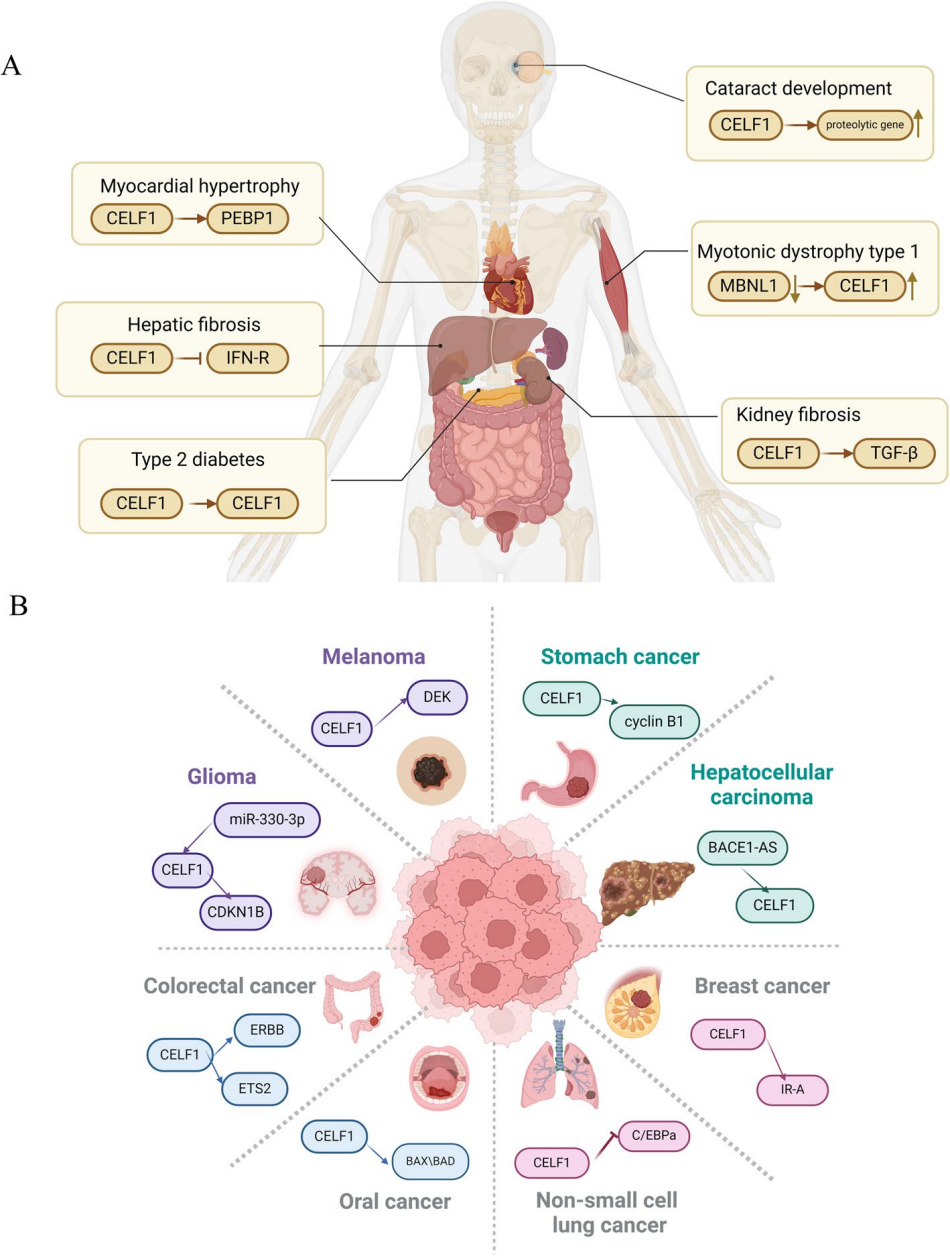
The association of CELF1 with diverse pathological conditions. **A** The function of CELF1 in non-neoplastic disorders. These encompass myotonic dystrophy type 1, myocardial hypertrophy, kidney fibrosis, hepatic fibrosis, cataract formation, and type 2 diabetes. **B** The function of CELF1 in cancer. These encompass melanoma, glioma, colorectal cancer, oral cancer, non-small cell lung cancer, breast cancer, hepatocellular carcinoma and stomach cancer

In recent years, extensive research has demonstrated a strong association between CELF1 and various human diseases. CELF1 has been shown to play a role in regulating the onset and progression of several cancers affecting different organs, including the oral cavity, liver, lung, and intestine. Its involvement in the pathogenesis of conditions such as restrictive muscular dystrophy, myocardial hypertrophy, and cataract formation has also been implicated. Therefore, understanding the relationship between CELF1 and these diseases, as well as the underlying regulatory mechanisms, is of great importance in the fields of disease prevention and therapeutic interventions (Fig. 4).

### CELF1 and non-cancer diseases

#### Myotonic dystrophy type 1

Myotonic dystrophy is an autosomal dominant genetic disorder that impacts various organ systems, encompassing the skeletal muscles, heart, and brain [28–31]. Specifically, DM1 arises from an aberrant expansion of microsatellite DNA, leading to the sequestration of non-coding RNA products by RNA splicing factors [32, 33]. This sequestration event results in the loss of functionality of RNA splicing factors, leading to widespread abnormalities in selective RNA splicing. Studies have demonstrated that CELF1 and MBNL1 exhibit antagonistic regulatory effects on post-transcriptional splicing and translation processes [34–37]. Previous reports have suggested that the primary mechanisms underlying splicing abnormalities in DM1 involve the downregulation of RNA splicing factor MBNL1 and its family members, along with the upregulation of an alternative RNA selective splicing factor, CELF1 [38–40].

#### Myocardial hypertrophy

The presence of ventricular hypertrophy has been identified as a predictor of heart failure and unfavorable cardiovascular outcomes [41, 42]. The modification of mRNA after transcription plays a pivotal role in controlling protein expression and the progression of cardiovascular diseases [27, 43, 44]. Previous studies have provided evidence indicating that CELF1 as a regulator of pathological myocardial hypertrophy and apoptosis by directly interacting with the 3' UTR of PEBP1, thereby impeding the activation of the MAPK signaling pathway [14]. This discovery opens up new possibilities for therapeutic interventions in treatment of hypertrophic cardiomyopathy and heart failure. Furthermore, several studies have shed light on a novel regulatory mechanism involving the interplay between *CELF1*, *HO-1*, and *CO* gene in hypertrophic cardiomyopathy (HCM). The regulatory role of CELF1 in HO-1, and its cardiovascular protective effects have revealed potential clinical applications and therapeutic strategies for managing HCM [45].

#### Kidney fibrosis

Chronic kidney disease (CKD) represents a significant health risk, and renal fibrosis is a key factor in the progression of CKD [46, 47]. The excessive activation of fibroblasts is a crucial initiating event in the development of renal fibrosis, which poses challenges for effective therapeutic interventions. Research investigating the involvement of CUGBP1 in fibroblast activation has demonstrated that reducing CELF1 expression significantly suppresses the downstream signaling pathway triggered by TGF-β. Moreover, the essential role of CELF1 in facilitating TGF-β-mediated activation of renal fibroblasts has been elucidated. A groundbreaking discovery has identified fraxinellone, a natural compound, as an effective agent for attenuating renal fibrosis. This compound achieves its inhibitory effect on renal fibroblast activation by downregulating CELF1 expression, offering a promising therapeutic approach for alleviating renal fibrosis [48]. Therefore, targeting the suppression of CELF1 expression emerges as a prospective strategy for mitigating renal fibrosis.

#### Hepatic fibrosis

Hepatic fibrosis is a pathological condition characterized by the excessive proliferation of connective tissue within the liver, resulted from various underlying causes [49]. The development of Hepatic fibrosis is intricately associated with the liver repair’s process. When injurious factors persist for an extended duration without resolution, it can progress to cirrhosis. CELF1 has been identified as a facilitator in the activation of hepatic stellate cells (HSCs) and the advancement of hepatic fibrosis by suppressing the expression of anti-fibrotic *IFN-γ* mRNA [50]. In studies involving mice, researchers have observed a targeted modulation of CELF1 expression in activated hematopoietic stem cells, aiming to mitigate liver fibrosis [51]. Consequently, CELF1 presents itself as a promising therapeutic target for the management of hepatic fibrosis and its associated conditions.

#### Cataract development

Cataracts are the primary cause of reversible visual impairment, and are characterized by the accumulation of newly generated lens cells onto existing cells [52]. This leads to the formation of cataracts, which are identified by lens discoloration and opacity due to hindered shedding and division of epithelial cells in the central region. The regulatory of CELF1, a critical post-transcriptional RBP, has been recognized in lens development [53, 54]. CELF1 modulates in alternative splicing of genes involved in DNA repair pathways by directly binding to their transcripts. Furthermore, CELF1 indirectly regulates the expression of proteolytic gene expression at the transcriptional level, playing critical role in lens development and the formation of cataracts [54–56].

#### Type 2 diabetes

CELF1 has been identified as a regulator of alternative splicing of insulin receptors, contributing to the promotion of insulin resistance [57–60]. Zhai et al. revealed that CELF1 is expressed in rodent islets and cell lines, with elevated levels observed within the islets of diabetic mice. CELF1 functions as a suppressor of insulin secretion in response to glucose and GLP-1 stimulation, exerting direct control over the expression of PDE3B by binding to its ATTTGTT sequence within the 3' UTR. The findings suggest that CELF1 plays a pivotal role for in the regulation of type 2 diabetes, indicating that targeting CELF1 may hold promise as a prospective therapeutic approach for combating to type 2 diabetes [59, 61].

### CELF1 and cancer

CELF1 has been found to be upregulated across various cancer types, and extensive investigations have revealed the mechanisms through which CELF1 operates in the context of cancer. Previous investigations have demonstrated that CELF1 protein acts as a central hub, controlling the translation and activation of genes involved in epithelial-mesenchymal transition (EMT) and tumor progresses. These findings emphasize the multifaceted involvement of CELF1 as a significant target within the context of cancer [62].

#### Melanoma

Melanoma, a highly aggressive malignancy that primarily originates from melanocytes, is commonly known as malignant melanoma [63]. The development of malignant melanoma is primarily attributed to DNA damage in melanocytes. Previous studies have shown that CELF1 is early induced as an RBP in melanoma cells and biopsies, indicating its potential as a key driver in cutaneous melanoma. Furthermore, the oncogene DEK has been identified as a signal amplifier in this context, contributing to the progression of melanoma [64].

#### Glioma

Glioma is the most commonly observed brain tumor and is characterized by high malignancy, frequent recurrence, intractable drug resistance, and challenging therapeutic strategies [65–67]. Elevated expression of CELF1 has been found to correlate with unfavorable overall survival outcomes in glioma patients. In an initial investigation using dual luciferase reporter gene assays, it was confirmed that miR-330-3p directly targets CELF1,resulting in the downregulation of CELF1 expression and subsequently inhibiting the proliferation and migration of glioma cells [68]. CELF1 promotes glioma cell proliferation by decreasing the expression levels of CDKN1B within the glioma microenvironment [69]. These findings suggest that CELF1 may serve as both a viable therapeutic target and a promising diagnosed biomarker for individuals afflicted with glioma.

#### Colorectal cancer

Colorectal cancer (CRC) is one of the most commonly diagnosed malignancies worldwide [70]. It arises from the progressive accumulation of genetic and epigenetic alterations. Hepatic metastasis is a frequently encountered site for the dissemination of colorectal cancer and contributes significantly to the mortality associated with this disease. Previous studies have confirmed the upregulation of CELF1 protein in both CRC tissues and CRC cell lines, indicating a correlation between heightened *CELF1* gene expression and the occurrence of liver metastasis [71]. CELF1 has the potential to facilitate the proliferation of colorectal cancer cells and their metastasis to liver through the ERBB signaling pathway [72]. Furthermore, it has been demonstrated that CELF1 enhances the migratory and invasive capacities of CRC cells, as well as their resistance to chemotherapy via interacting with *ETS2* mRNA, ultimately leading to increased expression of ETS2 [73].

#### Oral cancer

Oral squamous cell carcinoma (OSCC) refers to the transformation of squamous epithelium in the oral cavity into a malignant tumor [74]. Previous studies have consistently observed a significant increase in CELF1 expression in both OSCC tissues and cell lines [75]. The overexpression of CELF1 has been associated with the 3' UTR that encodes proapoptotic factors, namely BAX, BAD, and JunD, leading to reduced expression of apoptotic factors. This regulatory mechanism influences the proliferation and apoptosis of oral cancer cells [76].

#### Non-small cell lung cancer

Lung cancer is a significant global health concern and a leading cause of cancer-related deaths [77]. Understanding the mechanisms involved in the initiation and progression of non-small cell lung cancer (NSCLC), which is associated with poor prognosis and low survival rates, is crucial for the development of effective therapeutic interventions. Previous studies have shown that upregulation of CELF1 in NSCLC leads to the downregulation of the C/EBPa pathway, promoting cellular proliferation and suppressing apoptosis [78–81].

#### Breast cancer

Breast cancer, characterized by the uncontrolled proliferation of mammary epithelial cells, has a high incidence rate among women [82, 83]. The oncogenic role of the insulin receptor (IR) has been observed in diverse cancer types. The insulin receptor gene (*INSR*) undergoes selective alternative splicing, resulting in the generation of two distinct isoforms, namely IR-A and IR-B. Among these isoforms, it has been established that IR-A predominantly governs cellular proliferation [84–86]. CELF1, a splicing factor, is capable of recognizing the sequence within the 10th intron and 11th exon of INSR. It promotes the exclusion of exon 11, leading to the expression of IR-A in breast cancer cells [87]. Additionally, it has been observed that insulin stimulation enhances the carcinogenic regulatory effects of CELF1 in breast cancer cells. This insight into the interplay between CELF1 and the insulin receptor sheds light on potential mechanisms underlying breast cancer progression and offers avenues for further research into targeted therapies.

#### Hepatocellular carcinoma

Primary liver cancer accounts for approximately 7% of all tumor diseases, with hepatocellular carcinoma (HCC) being the most common subtype [88]. Despite being a well-known form of cancer, the underlying pathogenesis of HCC remains poorly understood [89]. HCC is associated with high malignancy, easy to recurrence, potential metastasis, and unfavorable prognosis [90, 91]. Therefore, early detection and diagnosis of HCC are essential to improve patient outcomes. The researchers have identified an association between long non-coding RNA *BACE1-AS*, *microRNA-377-3p*, and CELF1 in HCC. BACE1-AS induces the EMT and regulates the miR-377-3p/CELF1 axis, thereby promoting the invasion and metastasis of HCC cells [91, 92]. The findings hold promise for developing genetic diagnostic tools and improving early cancer detection methods. Moreover, the BACE1-AS/miR-377-3p/CELF1 regulatory axis identified in HCC may serve as a therapeutic target for RNA interference-based interventions. the of BACE1-AS/miR-377-3p/CELF1 axis in HCC may serve as a therapeutic target for RNA interference-based interventions.

#### Gastric cancer

Gastric cancer is the second leading cause of cancer-related deaths, presenting a significant management challenge. Traditional approaches such as radiotherapy and chemotherapy have been insufficient in controlling the progression and metastasis of this malignancy [93, 94]. Wang et al.’s study demonstrated a substantial increase in CELF1 expression in gastric cancer tissues compared to adjacent normal tissues. Upon downregulation of CELF1, a reduction in colony formation capacity were observed, along with the downregulation of cyclin B1 and cyclin D1, key signaling molecules involved in regulating cell cycle progression. The findings underscore the essential role of CELF1 in gastric cancer cell proliferation, highlighting the potential of RNA interference-mediated CELF1 silencing as a promising therapeutic strategy for gastric cancer [95].

## Molecular mechanisms of action of CELF1

**Fig. 5:**
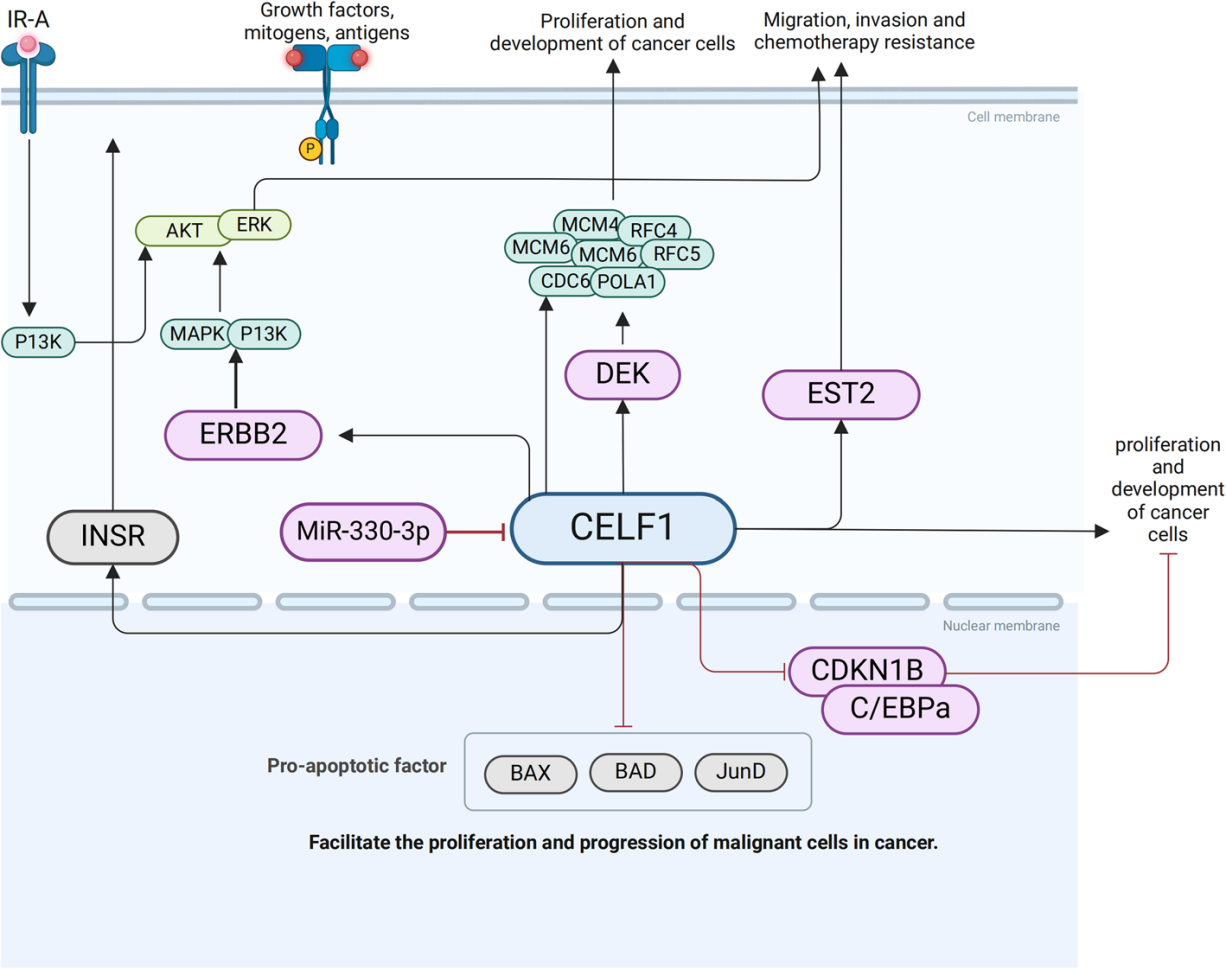
The molecular mechanism underlying the involvement of CELF1 in cancer

CELF1 is involved in the modulation of multiple signaling pathways, and recent investigations into the AKT/ERK pathway governed by CELF1 have provided clear insights. Several studies have demonstrated that increased CELF1 expression in mammalian cells hampers the transcriptional activity of *CDKN1B*, *BAX*/*BAD/JunD*, and *C/EBPa*. Conversely, downregulation of CELF1 has been found to alleviate deficiencies in transcriptional inhibition and plays a significant role in various cancer types. In the majority of cancers, CELF1 promotes cancer cell proliferation, metastasis, and invasion by activating downstream pathways including AKT/ERK and ETS2 (Table 1 and Fig. 5). These pathways are known to be involved in cell survival, growth, and migration. The downregulation of CELF1 can disrupt these signaling cascades and potentially impede the progression of cancer. Understanding the role of CELF1 in cancer development and its interaction with signaling pathways provides valuable insights into potential therapeutic strategies for cancer treatment.

**Table 1:** The functional roles of CELF1 in cancer

Genes	Pathways/binding site	Target genes	Functions	Refs.
CELF1	3 ‘UTR GREs	DEK	Stabilize DEKmRNA and amplify carcinogenic signaling	[64]
	Untranslated and overexpressed regions	CDKN1B	Inhibit the expression of CDKN1B and promote the proliferation of cancer cells	[69]
	ERBB2/MAPK/P13K	AKT/ERK	Promote the proliferation of cancer cells	
	mRNA 3 ‘UTR	ETS2	Promote the proliferation、metastasis and invasion of cancer cells	[73]
	mRNA 3 ‘UTR	BAX/BAD/JunD	Inhibit the expression of BAX/BAD/JunD and promote the proliferation of cancer cells	[76]
	C/EBPa	C/EBPa	Inhibit the expression of C/EBPa and promote the proliferation of cancer cells	[78–81]
	INSR shear	IR-A	Promote IR-A expression, and influences carcinogenic signaling through the insulin-signaling pathway of breast cancer	[87]
MiR-330-3p	mRNA 3 ‘UTR	CELF1	Down-regulating the expression of CELF1 inhibited the proliferation and migration of glioma cells	[68]
BACE1-AS	miR-377-3p	miR-377-p/CELF1 axis	Promoting the invasion and metastasis of liver cancer cells	[92]

### DEK

DEK (DEK Proto-Oncogene) is classified as a gene involved in encoding a protein with a functional role in cellular processes related to cancer development. DEK has been implicated in the pathogenesis of various diseases, including acute myeloid leukemia and iridocyclitis. CELF1 has been implicated as a key driver in skin melanoma and functions as an oncogene by amplifying the signaling effect of DEK SpecificallyCELF1 achieves this by binding to the GU-rich 3' UTR region of DEK mRNA, which leads to an extended half-life of DEK mRNA. The oncogenic activity of DEK facilitated by CELF1, then exerts control over the mRNA and protein expression levels of critical DNA replicators, including MCM4, MCM6, RFC4, RFC5, CDC6, and POLA1. This regulatory mechanism ultimately governs the progression and development of melanoma [64]. These findings highlight the intricate interplay between CELF1, DEK, and DNA replicators in melanoma pathogenesis, shedding light on potential therapeutic targets for the disease.

### CDKN1B

CDKN1B, also known as p27, is a well-stablished tumor suppressor that plays a crucial role in cell proliferation by inhibiting cyclin-dependent activity [96, 97]. Its interaction with cyclin complexes and CDK2 serves to halt the progression of the cell cycle from G1 to S phase. In glioma cells, CELF1 is bound to suppress the expression of endogenous CDKN1B and attenuates translation initiation via interaction with the overexpression region of CDKN1B. This process ultimately facilitates the proliferation of glioma cells [69].

### EST2

V-ets avian erythroblastosis virus E26 oncogene homolog 2 (ETS2) is an evolutionarily conserved transcription factor that belongs to the ETS family. ETS factors, including ETS2, regulate specific genes that play crucial roles in various cellular processes, such as cell proliferation, apoptosis, differentiation, lymphocyte development, angiogenesis, and invasiveness. It have been observed that CELF1 induces the upregulation of ETS2 by binding to it, and thus enhances migration, invasion, and chemotherapy resistance in CRC cells [73].

### BAX/BAD/JunD

The BCL2 family plays a critical role in regulating apoptosis. BCL2, in particular, exerts an anti-apoptotic function by interacting with pro-apoptotic members such as BAX and BAK, thereby preventing the initiation of cytochrome C release and inhibiting apoptosis [98]. Additionally, the protein BAD, apart from its pro-apoptotic role, sequesters BCL2 and hinders its interaction with BAX and BAK, further modulating apoptotic pathways [99]. Furthermore, JunD acts as a multifunctional transcription factor that modulates various target genes involved in apoptosis, angiogenesis, and cellular differentiation through its functions of activation or inhibition. Notably, CELF1 has been found to regulate the proliferation and apoptosis of oral cancer cells by downregulating the expression of pro-apoptotic factors, namely BAX, BAD, and JunD [76]. These interactions underscore the intricate regulatory mechanisms governing apoptotic pathways and their dysregulation in the context of cancer.

### MiR-330-3p

MicroRNAs (miRNAs) are a class of widely conserved, non-coding small RNA molecules that are approximately 20 nucleotides in length. They have been found to play a role in tumor development and carcinogenesis in glioma tissues and cells [100]. In the case of miR-330-3p, it has been observed that it is negatively correlated with CELF1 expression. By targeting the 3'UTR of CELF1, miR-330-3p downregulates the expression of CELF1. This downregulation leads to the inhibition of proliferation and migration of glioma cells. These findings suggest that miR-330-3p may serve as a potential therapeutic target for glioma treatment by modulating CELF1 expression and its associated cellular processes [68].

## CELF1 regulators

### Dimethylisoquinolines

**Fig. 6:**
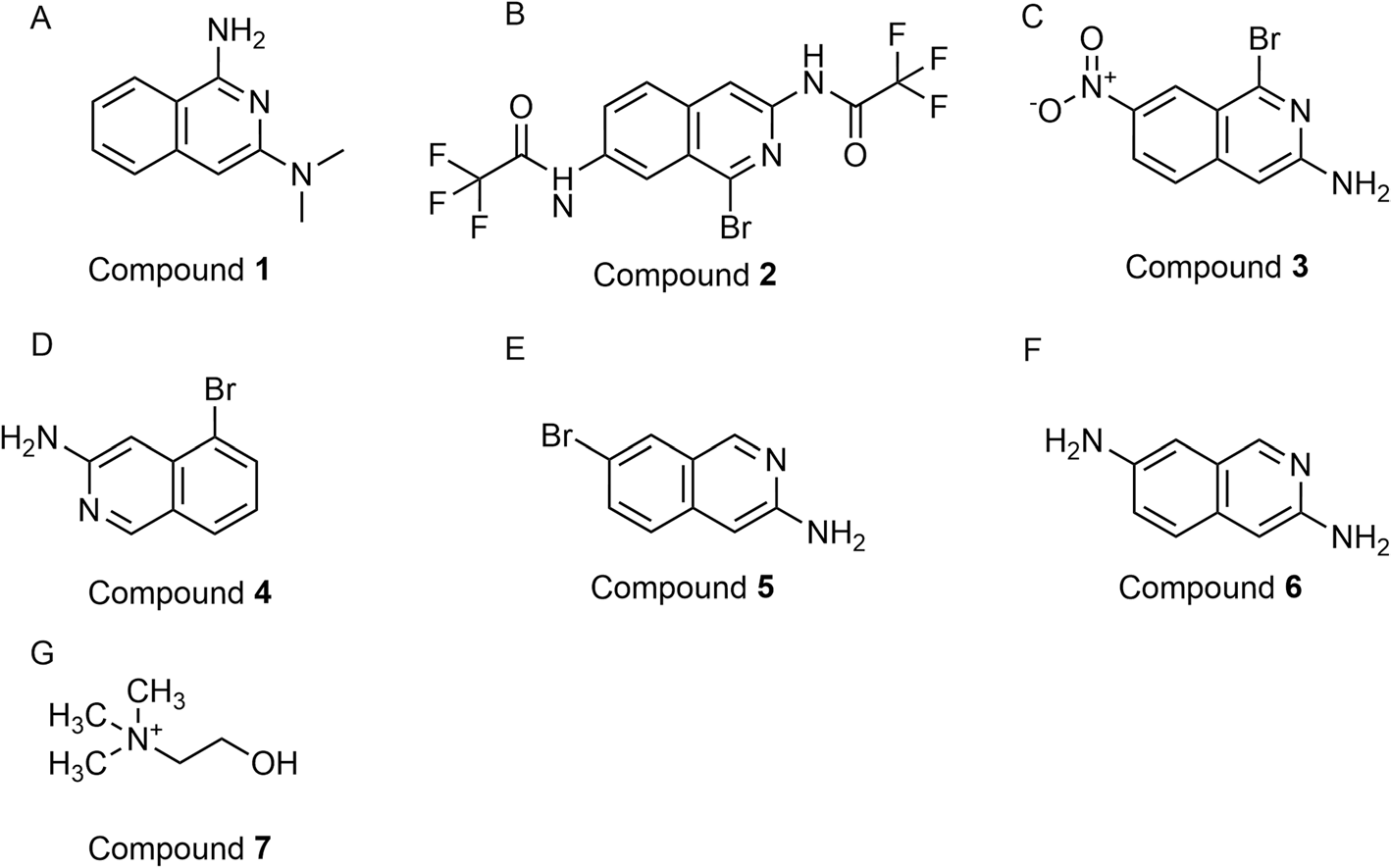
The chemical structure of Compounds acting as regulators of CELF1. **A** Compound **1**. **B**–**F** The derivative of compound** 1**. **G** Choline

**Fig. 7:**
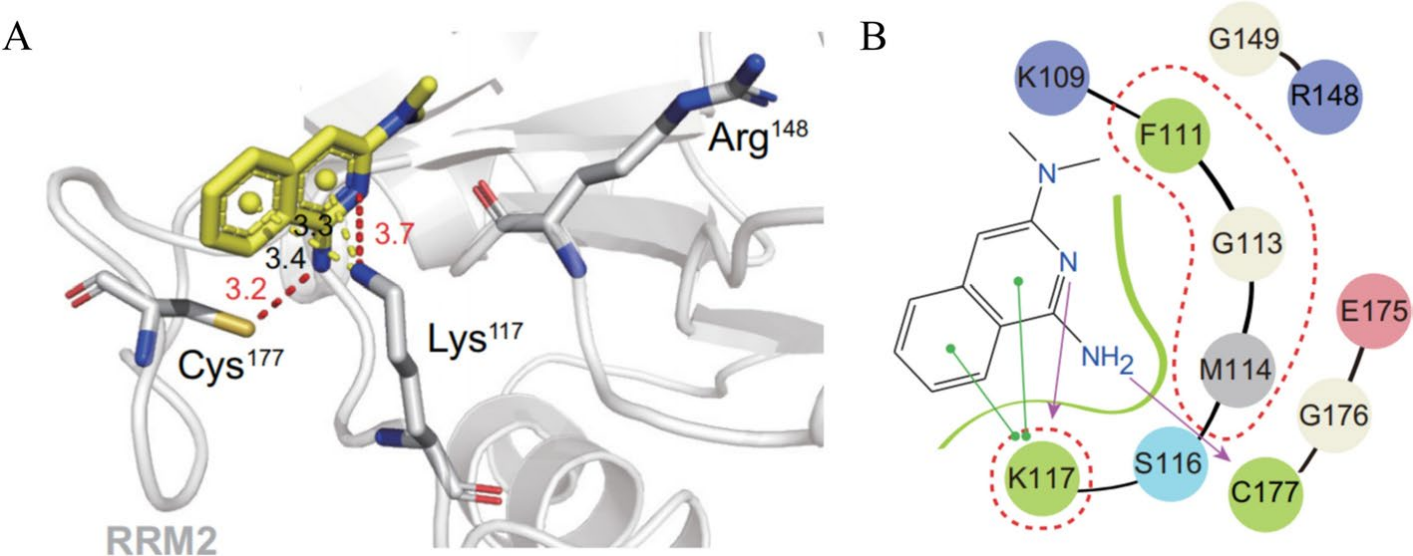
Illustration depicting the binding of compound **1** to CELF1. **A** The docking mode of compound **1** with the interaction residues (Lys117, Cys177) and corresponding distances. **B** 2D representation of compound **1** binding with RRM2. The permission to use these figures for academic purposes have been obtained from Refs. [50, 101]

RNA-binding proteins have garnered considerable attention as potential therapeutic targets. However, the majority of RBPs are considered "undruggable" due to the lack of well-defined binding domains. Therefore, a strategy has emerged to disrupt the interactions between RBPs and RNA by targeting their RNA-binding activity, aiming to impede their function. Previous studies have shown that CELF1, an RBP consisting of three RRMs, exhibits a specific affinity for guanosine-rich elements. The researchers sought to selectively disrupt the interaction between RRM1/2 and UGUU elements to inhibit CELF1's RNA-binding ability. The crystal structure of CELF1 was utilized by the researchers to conduct a screening of inhibitors targeting RNA binding activity. From a pool of 10,000 compounds, the top 90 molecules were selected for subsequent biochemical analysis. Compound **1** exhibited the most pronounced competitive activity as determined through a FP assay. To validate this finding, an electrophoretic mobility transfer assay (EMSA) was employed, revealing that compound** 1** effectively disrupted the interaction between RRM1/2/3 and RNA. Importantly, compound **1** did not impede the binding of HUL-RNA, nor did it impact the stability of MMP9 and VEGFA mRNA. Consequently, compound **1** was deemed worthy of further investigation (Fig. 6A). Subsequent analyses elucidated that **1** predominantly binds to the K117 residue of CELF1, effectively competing with GU-rich RNA, and thereby disintegrating the RRM1/2/3-RNA interaction (Fig. 7A, B). Compound **1** impedes CELF1-mediated *IFN-γ* mRNA degradation and effectively regulates stellate cell activation. In an in vivo mouse model of liver fibrosis induced by CTC (carbon tetrachloride) has shown that compound **1** has a mitigating effect on liver fibrosis. Further studies involved screening derivatives (**2**–**5**) of compound **1** (Fig. 6B–F), and among them, compound **6** was found to selectively inhibit the RNA binding function of CELF1 (Fig. 6F). These encouraging findings highlight the potential of developing CELF1 RNA-binding inhibitors as a novel therapeutic strategy for liver fibrosis [50, 101].

### Choline

The recent investigations concerning the interplay between choline and CELF1 have brought to light crucial insights into the potential therapeutic implications of choline in modulating CELF1 expression. These studies emphasize the importance of adequate duration and dosage of choline to effectively suppress CELF1 expression (Fig. 6G). The modulation of CELF1 expression by choline likely implicates post-transcriptional mechanisms, which may include the recruitment of CELF1 mRNA into processing bodies. Furthermore, it is suggested that choline-induced alterations in the microRNA pool play a significant role in this pathway. While the precise microRNAs implicated have yet to be identified. The evidence indicates a competitive relationship between OOLE (oxidized omega-6 linoleic acid esters) and choline, which may influence the fate of CELF1 mRNA, leading to either translation in the ribosome or degradation in the processing bodies. Previous research has demonstrated that supplementary choline can mitigate OOLE-induced enterocyte apoptosis both in vivo and in vitro, potentially through the inhibition of CELF1 translation and subsequent suppression of the CELF1/AIF pathway. These findings provide empirical evidence supporting the therapeutic potential of choline supplementation within clinical Total Parenteral Nutrition (TPN) protocols for managing TPN-induced intestinal atrophy. Overall, these investigations shed light on the intricate regulatory mechanisms involving choline and CELF1, offering potential avenues for therapeutic interventions in conditions such as TPN-induced intestinal atrophy. Further research in this area could elucidate the precise molecular mechanisms and identify specific microRNAs involved, paving the way for targeted therapeutic strategies leveraging the interplay between choline and CELF1 [102].

## Conclusions and prospects

As a member of the RNA-binding protein family, increasing evidence supports CELF1 as a key regulatory factor in transcriptional regulation, mRNA splicing, cell proliferation, and cell cycle progression, playing important roles in maintaining homeostasis, development, and cancer. Multiple studies have suggested that CELF1 is a potential therapeutic target in cancer. Although there have been numerous reports on the role of CELF1 in cancer progression and development, further research is still needed to construct a more precise CELF1-targeted regulatory network and explore its functions in various types of cancer. This may involve comprehensive exploration of CELF1's transcriptional regulation, including the identification of key splicing targets and the coordination of protein–protein interactions involved in these regulatory processes. Additionally, while some studies have identified associations between CELF1 and non-cancerous diseases such as diabetes [103, 104], Alzheimer's disease [105–108], and obesity [103, 105, 109], the exact molecular mechanisms underlying its regulatory role in these diseases still require further investigation.

A more critical issue is the observed functional redundancy. The sequence conservation between the connecting region of RRM2 and RRM3 in CELF1's domain is low, while RRM3 shows higher conservation. However, in most studies, RRM3 seems to be dispensable. The specific function of RRM3 remains unclear and can only be scientifically explained through further experimental research using highly specific tools. Furthermore, while previous studies have mainly focused on the association between CELF1 and cancer, the more precise and in-depth biological functions of CELF1 have been less explored. In fact, CELF1 plays important roles in selective splicing, embryonic development, and activation of immune cells. To comprehensively understand the biological functions of CELF1, key data are needed, including the roles and precise enumeration of mRNA variants and protein isoforms in various cellular and tissue environments. Further research is also needed to elucidate the specific mechanisms of action of CELF1's RRMs and their interactions with RNA targets, as well as the exact molecular mechanisms regulating their processing and translation. Additionally, the expression patterns of CELF1 mRNA variants and protein isoforms in specific cell types, different cell cycles, or specific stages of development have not been widely studied. Understanding the differential expression patterns of CELF1 in different environments can provide insights into its functional diversity and regulatory roles in various biological processes.

Previous studies have shown that CELF1 is an attractive target for the development of novel molecular-targeted cancer therapies. However, research on CELF1 inhibitors has been lacking for a long time, with only a few publications reporting some indirect modulators. Recently, exciting clues have emerged in CELF1 research, discovering a small molecule inhibitor (compound **1**) that effectively inhibits CELF1 binding activity and is considered a potential therapeutic approach for liver fibrosis. However, no CELF1 inhibitors have been approved by the FDA, and there are no clinical trials, making CELF1 still an "undruggable" target. Further research and support are needed to better achieve the discovery and development of CELF1 drugs. Currently, research on RNA-binding protein inhibitors is still in its early stages. High-throughput screening can be utilized to screen more compound libraries in search of high-affinity compounds targeting CELF1 as inhibitors. Additionally, RNA interference techniques can be used to find siRNAs or shRNAs to inhibit CELF1 expression.

In conclusion, CELF1 holds great research potential as a potential target in cancer. Although there is still a long way to go in the study and application of CELF1 inhibitors, further understanding of CELF1's mechanisms and inhibitors is necessary due to its rich network of targeted effects in multiple cancers. Furthermore, simultaneous efforts should be made to further elucidate the specific biological functions of CELF1 in order to advance precise treatments for CELF1-driven cancers in the clinical setting.

## Data Availability

Not applicable.

## Abbreviations

RBPs: RNA-binding proteins
RBDs: RNA-binding domains
CELF1: CUG-BP, Elav-like family 1
UTRs: Untranslated regions
DM1: Myotonic dystrophy type 1
HCM: Hypertrophic cardiomyopathy
CKD: Chronic kidney disease
HSCs: Hepatic stellate cells
EMT: Epithelial-mesenchymal transition
CRC: Colorectal cancer
OSCC: Oral squamous cell carcinoma
NSCLC: Non-small cell lung cancer
IR: Insulin receptor
INSR: Insulin receptor gene
HCC: Hepatocellular carcinoma
ETS2: E26 oncogene homolog 2
miRNAs: MicroRNAs
EMSA: Electrophoretic mobility transfer assay
CTC: Carbon tetrachloride
OOLE: Oxidized omega-6 linoleic acid esters
TPN: Total parenteral nutrition
